# SITC/iSBTc Cancer Immunotherapy Biomarkers Resource Document: Online resources and useful tools - a compass in the land of biomarker discovery

**DOI:** 10.1186/1479-5876-9-155

**Published:** 2011-09-19

**Authors:** Davide Bedognetti, James M Balwit, Ena Wang, Mary L Disis, Cedrik M Britten, Lucia G Delogu, Sara Tomei, Bernard A Fox, Thomas F Gajewski, Francesco M Marincola, Lisa H Butterfield

**Affiliations:** 1 Society for Immunotherapy of Cancer, Milwaukee, WI, USA; 2 Infectious Disease and Immunogenetics Section (IDIS), Department of Transfusion Medicine, Clinical Center and Trans-NIH Center for Human Immunology (CHI), National Institutes of Health, Bethesda, MD, USA; 3 Department of Internal Medicine (DiMI), University of Genoa, and Department of Medical Oncology, National Cancer Research Institute of Genoa, Genoa, Italy; 4 Tumor Vaccine Group, Division of Oncology, University of Washington, Seattle, WA, USA; 5 Department of Medicine, University Medical Center of the Johannes Gutenberg-University, and Ribological GmbH, Mainz, Germany, Department of Internal Medicine, University Medical Center of the Johannes Gutenberg-University, and Clinical Development, BioNTech AG, Mainz, Germany; 6 University of Sassari, Department of Drug Sciences, Sassari, Italy; 7 Division of Surgical, Molecular and Ultrastructural Pathology, Department of Oncology, University of Pisa and Pisa University Hospital, Pisa, Italy; 8 Laboratory of Molecular and Tumor Immunology, Earle A. Chiles Research Institute, Robert W. Franz Cancer Center, Providence Portland Medical Center, Portland, OR, USA; 9 Section of Hematology/Oncology, Department of Pathology and Department of Medicine, University of Chicago, Chicago, IL, USA; 10 Departments of Medicine, Surgery and Immunology, University of Pittsburgh, Pittsburgh, PA, USA

## Abstract

Recent positive clinical results in cancer immunotherapy point to the potential of immune-based strategies to provide effective treatment of a variety of cancers. In some patients, the responses to cancer immunotherapy are durable, dramatically extending survival. Extensive research efforts are being made to identify and validate biomarkers that can help identify subsets of cancer patients that will benefit most from these novel immunotherapies. In addition to the clear advantage of such predictive biomarkers, immune biomarkers are playing an important role in the development, clinical evaluation and monitoring of cancer immunotherapies. This Cancer Immunotherapy Resource Document, prepared by the Society for Immunotherapy of Cancer (SITC, formerly the International Society for Biological Therapy of Cancer, iSBTc), provides key references and online resources relevant to the discovery, evaluation and clinical application of immune biomarkers. These key resources were identified by experts in the field who are actively pursuing research in biomarker identification and validation. This organized collection of the most useful references, online resources and tools serves as a compass to guide discovery of biomarkers essential to advancing novel cancer immunotherapies.

## Introduction

Immunotherapy has emerged as an important treatment strategy for patients with cancer. With several recent approvals by the U.S. Food and Drug Administration (FDA), cancer immunotherapy has become the latest addition to the toolbox of effective cancer treatments that includes chemotherapy, signal transduction inhibitors, anti-angiogenic agents, radiotherapy, and surgery.

Successful development and testing, regulatory approval and clinical application of cancer immunotherapies require the identification and validation of biomarkers of efficacy. The importance of reliable biomarkers to guide immune-based and personalized cancer therapies is clear. Biomarkers can aid in early disease diagnosis, help clinicians identify patients most likely to benefit from these expensive treatments, and facilitate drug discovery, development and biological/clinical evaluation of cancer immunotherapies.

For over twenty-five years the Society for Immunotherapy of Cancer (SITC; formerly the International Society for Biological Therapy of Cancer, iSBTc) has advanced the science, development and application of biological therapy/immunotherapy of cancer. The society has long recognized the importance of biomarkers for cancer immunotherapy, which has been the focus of a number of SITC/iSBTc symposia and workshops [1-5], and has published recommendations [6] and summaries [7-10].

To support the efforts of investigators involved in research to identify and validate biomarkers for cancer immunotherapy, the authors and members of the SITC Biomarkers Taskforce have identified key biomarker references and online resources and organized these into this SITC/iSBTc Cancer Immunotherapy Biomarkers Resource Document. This document provides an overview of suggested publications and resources for studies on biomarkers for cancer immunotherapy. This resource document is divided into two sections: Part I: Immunotherapy Biomarker References; and Part II: High Throughput and New Technologies for Biomarker Discovery: Arrays, Platforms, Tools for The Bench and Online Resources. While many important references and resources in the field are included in this document, it does not intend to represent an exhaustive list of all relevant publications, products or resources in the growing, and important field of immune biomarkers. A comprehensive list of online tools for bioinformatics and molecular biology research is available from the Bioinformatics Links Directory [11,12].

A draft of the present document was originally provided to attendees of the SITC/iSBTc Symposium on Immuno-Oncology Biomarkers, 2010 and Beyond: Perspectives from the iSBTc Biomarker Task Force [1], which was held September 30, 2010 at the National Institutes of Health in conjunction with the society's 25th Annual Meeting. Following the symposium, the draft document was posted on the society's website for open comment. The comments were reviewed by the authors and incorporated into this manuscript. The references and online resources are organized as outlined in Table 1.

**Table 1:** Structure of the Biomarker References and Online Resources Provided

**Part I. Immunotherapy Biomarker References**

**1. Guideline and Meeting Reports**

**2. Clinical Trials**
A. Recent Immunotherapy Trials in Cancer Patients - Positive Randomized Phase III Studies B. Emerging Biomarkers for Immunotherapy of Cancer - Clinical Trials C. Correlation between Immune Response and Clinical Outcome in Cancer Patients; Positive Studies

**3. Gene Expression Profiling in Cancer Immunotherapy**

**4. Useful Reviews**

**5. Bioinformatics for Biomarkers Discovery**

**6. New Technologies for Biomarkers Discovery**

**7. Standardization and Harmonization of Sample Collection and Use of Immunological Assays**
A. Processing, Storage and Shipping of Blood Samples and Serum for Immunological Studies - Technical Considerations B. Cellular Immunotherapy: Characterization of Cellular Products C. Assay Standardization and Harmonization D. Assays for Determination of Antitumor Immune-Response in Clinical Trials

**8. Reporting Biomarkers Data in Publications**

**Part II. High Throughput and New Technologies for Biomarker Discovery: Arrays, Platforms, Tools for The Bench and Online Resources**

**1. Genomic Biomarkers Discovery**
A. Single Nucleotide Polymorphisms (SNPs) Arrays B. Comparative Genomic Hybridization (CGH) Arrays C. Mitochondrial Genome Arrays

**2. Epigenomic Biomarkers Discovery**
A. Methylation Arrays B. microRNA (miRNA) Arrays C. Chromatin Immunoprecipitation (ChIP) Arrays (ChIP on chip)

**3. Transcriptomic Biomarkers Discovery**
A. Expression Arrays B. Quantitative Assays

**4. Proteomic Biomarkers Discovery**
A. Protein and Phosphoprotein Assays B. Multicolor Cytometric Systems

**5. Next Generation Sequencing: Whole Genomic and Transcriptomic Sequencing Systems**

**6. Software and Tools for Data Analysis**
A. Microarrays/Sequencing Data Analysis B. Next Generation Sequencing Data Analysis C. Multicolor Cytometric Data Analysis

**7. Software and Tools for Function and Pathway Analysis**

**8. siRNA Libraries**

**9. Public Databases**

**10. Tools for the Bench and Other Useful Websites**
A. Primer Design Software B. Transcription Factors Binding Sites Prediction Software C. Design of Antisense Oligonucleotides, Nucleic Acid Probes, siRNA Software D. miRNA Prediction E. Alternative Splicing Analysis F. Linkage Disequilibrium Analysis G. Analysis Support, Laboratory Optimization and Other Useful Websites H. Nanotechnology I. Clinical Trials Registries

## Part I. Immunotherapy Biomarker References

### 1. Guideline and Meeting Reports

#### Recommendations from the iSBTc/FDA/NCI Workshop on Immunotherapy Biomarkers

Butterfield LH, Disis ML, Fox BA, et al.: A systematic approach to biomarker discovery; Preamble to "The iSBTc-FDA Taskforce on Immunotherapy Biomarkers". J Transl Med 6:81, 2008

Butterfield LH, Palucka AK, Britten CM et al:: Recommendations from the iSBTc-SITC/FDA/NCI Workshop on Immunotherapy Biomarkers, Clin Cancer Res 17:3064-3076, 2010

#### 2009 Report of the US - Japan Workshop on Immunological Biomarkers in Oncology

Tahara H, Sato M, Thurin M, et al.: Emerging concepts in biomarker discovery; The US-Japan workshop on immunological molecular markers in oncology. J Transl Med 7:45, 2009

#### 2010 Guidelines of the NCI-Investigational Drug Steering Committee (IDSC) Biomarker Task Force

Dancey JE, Dobbin KK, Groshen S, et al.: Guidelines for the development and incorporation of biomarker studies in early clinical trials of novel agents. Clin Cancer Res 16:1745-1755, 2010

#### 2010 Report of the Translational Research Cancer Centers Consortium (TrC3) Immunotherapy Network Annual Meeting

Lesinski GB, Carson WE, Repasky EA, et al.: Meeting Report: The 13th Annual Meeting of the Translational Research Cancer Centers Consortium (TrC3); Immune Suppression and the Tumor Microenvironment, Columbus, Ohio; March 1-2, 2010. J Immunother, 2010

#### 2008 Report on EU-USA Workshop on System Biology in Cancer Research

Aebersold R, Auffray C, Baney E, et al.: Report on EU-USA workshop: how systems biology can advance cancer research (27 October 2008). Mol Oncol 3:9-17, 2009

#### 2007 AACR-FDA-NCI Cancer Biomarkers Collaborative Consensus Report

Khleif SN, Doroshow JH, Hait WN: AACR-FDA-NCI Cancer Biomarkers Collaborative Consensus Report: Advancing the Use of Biomarkers in Cancer Drug Development. Clin Cancer Res 16:3299-3318, 2010

#### 2005 Report on Workshop on Cancer Biometrics Held at NIH Masur Auditorium

(This manuscript also includes a brief description of high throughput technologies)

Lotze MT, Wang E, Marincola FM, et al.: Workshop on cancer biometrics: identifying biomarkers and surrogates of cancer in patients: a meeting held at the Masur Auditorium, National Institutes of Health. J Immunother 28:79-119, 2005

#### 2002 Report on Workshop on Immunologic Monitoring of Cancer Vaccine Trials

Keilholz U, Weber J, Finke JH, et al.: Immunologic monitoring of cancer vaccine therapy: results of a workshop sponsored by the Society for Biological Therapy. J Immunother 25:97-138, 2002

### 2. Clinical Trials

#### A. Recent Immunotherapy Trials in Cancer Patients - Positive Randomized Phase III Studies

Examples

##### gp100:209-217 (210M) Peptide Followed by High-Dose IL-2 - Metastatic Melanoma

Schwartzentruber DJ, Lawson D, Richards J, et al: gp100 peptide vaccine and interleukin-2 in patients with advanced melanoma. N Eng J Med 364:2119-27, 2011

##### Anti-GD2 Antibody with GM-CSF, Interleukin-2, and Isotretinoin - High-Risk Neuroblastoma

Yu AL, Gilman AL, Ozkaynak MF, eta al.: Anti-GD2 antibody with GM-CSF, interleukin-2, and isotretinoin for neuroblastoma. N Engl J Med. 363:1324-34, 2010

##### Ipilimumab and Dacarbazine in Metastaic Melanoma Patients - Untreated Metastatic Melanoma

Robert C, Thomas L, Bondarenko I, et al.: Ipilimumab plus dacarbazine for previously untreated metastatic melanoma. N Engl J Med 364:2517-26, 2011

##### Ipilimumab in Metastatic Melanoma Patients - Previously Treated Metastatic Melanoma

Hodi FS, O'Day SJ, McDermott DF, et al.: Improved survival with ipilimumab in patients with metastatic melanoma. N Engl J Med 363: 711-23, 2010

##### Sipuleucel-T Immunotherapy - Metastatic Prostate Cancer

Kantoff PW, Higano CS, Shore ND, et al.: Sipuleucel-T immunotherapy for castration-resistant prostate cancer. N Engl J Med 363:411-422, 2010

##### Interferon α Adjuvant Treatment - High Risk Melanoma

Eggermont AMM, Suciu S, Santinami M, et al.: Adjuvant therapy with pegylated interferon alfa-2b versus observation alone in resected stage III melanoma: final results of EORTC 18991, a randomised phase III trial. Lancet 372:117-126, 2008

##### Idiotype Vaccine Therapy - Complete Remission Follicular Lymphoma

Schuster SJ, Neelapu SS, Gause BL, et al: Vaccination with patient-specific tumor-derived antigen in first remission improves disease-free survival in follicular lymphoma. J Clin Oncol 20: 2787-2794, 2011

#### B. Emerging Biomarkers for Immunotherapy of Cancer - Clinical Trials

Examples

##### Parameter: HLA Cw*06 Allele

##### Setting: Interferon α Adjuvant Treatment - High-Risk Melanoma

Gogas H, Kirkwood JM, Falk CS, et al.: Correlation of molecular human leukocyte antigen typing and outcome in high-risk melanoma patients receiving adjuvant interferon. Cancer 116:4326-33, 2010

##### Parameter: CCR5 and CXCR3 Polymorphisms and Gene Expression

##### Setting: Adoptive Therapy - Metastatic Melanoma

Bedognetti D, Uccellini L, Wang, et al.: Evaluation of CXCR3 and CCR5 polymorphisms and gene-expression as predictive biomarkers of clinical response to adoptive therapy in melanoma patients. J Immunother 33:860, 2010 (Meeting Abstract)

##### Parameter: Differentiated Effector Phenotype, ''B and T Lymphocyte Attenuator'' (BTLA) Expression in CD8+ TIL

##### Setting: Adoptive Therapy - Metastatic Melanoma

Laszlo G. Radvanyi, Chantale Bernatchez, Minying Zhang, et al. Adoptive T cell therapy for metastatic melanoma: The MD Anderson experience. J Immunother 33:863, 2010 (Meeting Abstract)

##### Parameter: VEGF Serum Level

##### Setting: IL-2 Therapy - Metastatic Melanoma

Sabatino M, Kim-Schulze S, Panelli MC, et al.: Serum vascular endothelial growth factor and fibronectin predict clinical response to high-dose interleukin-2 therapy. J Clin Oncol 27:2645-2652, 2009

##### Parameter: Treg Numbers

##### Setting: hTERT Pulsed DCs Therapy - Metastatic Solid Tumors

Aloysius MM, Mc Kechnie AJ, Robins RA, et al.: Generation in vivo of peptide-specific cytotoxic T cells and presence of regulatory T cells during vaccination with hTERT (class I and II) peptide-pulsed DCs. J Transl Med 7, 2009

##### Parameter: CD54 Expression by APC

##### Setting: Sipuleucel-T Cellular Immunotherapy - Metastatic Prostate Cancer

Higano CS, Schellhammer PF, Small EJ, et al.: Integrated data from 2 randomized, double-blind, placebo-controlled, phase 3 trials of active cellular immunotherapy with sipuleucel-T in advanced prostate cancer. Cancer 115:3670-3679, 2009

##### Parameter: Delta32 CCR5 Polymorphism

##### Setting: Immunotherapy (IL2/IFNα ± Chemotherapy/Vaccination) - Metastatic Melanoma

Ugurel S, Schrama D, Keller G, et al.: Impact of the CCR5 gene polymorphism on the survival of metastatic melanoma patients receiving immunotherapy. Cancer Immunol Immunother 57:685-691, 2008

##### Parameter: Telomere Length

##### Setting: Adoptive Therapy - Metastatic Melanoma

Dudley ME, Yang JC, Sherry R, et al.: Adoptive cell therapy for patients with metastatic melanoma: evaluation of intensive myeloablative chemoradiation preparative regimens. J Clin Oncol 26:5233-5239, 2008

##### Parameter: CTLA-4 Polymorphisms

##### Setting: Anti-CTLA-4 Therapy - Metastatic Melanoma

Breunis WB, Tarazona-Santos E, Chen R, et al.: Influence of cytotoxic T lymphocyte-associated antigen 4 (CTLA4) common polymorphisms on outcome in treatment of melanoma patients with CTLA-4 blockade. J Immunother 31:586-9, 2008

##### Parameter: CCL5, CCL11, IFN-γ, ICOS, CD20

##### Setting: MAGEA3-Based Vaccination - Metastatic Melanoma

Louahed J, Gruselle O, Gaulis S, et al.: Expression of defined genes identified by pretreatment tumor profiling: Association with clinical responses to the GSK MAGE- A3 immunotherapeutic in metastatic melanoma patients (EORTC 16032-18031). J Clin Oncol 26:9045, 2008 (Meeting Abstract)

##### Parameter: Fc Gamma Receptor Polymorphisms

##### Setting: Immuno (Trastuzumab) Chemotherapy - Metastatic Breast Cancer

Musolino A, Naldi N, Bortesi B, et al.: Immunoglobulin G fragment C receptor polymorphisms and clinical efficacy of trastuzumab-based therapy in patients with HER-2/neu-positive metastatic breast cancer. J Clin Oncol 26:1789-1796, 2008

##### Parameter: Phosphorylated - STAT-1/STAT-3 Ratio

##### Setting: Neoadjuvant IFN-α Therapy - Stage IIIB Melanoma

Wang WJ, Edington HD, Rao UNM, et al.: Modulation of signal transducers and activators of transcription 1 and 3 signaling in melanoma by high-dose IFN alpha 2b. Clin Cancer Res 13:1523-1531, 2007

##### Parameter: IL 1β, IL-1α, IL-6, TNF-α, CCL3, CCL4

##### Setting: Adjuvant IFN-α Therapy - High Risk Melanoma

Yurkovetsky ZR, Kirkwood JM, Edington HD, et al Multiplex analysis of serum cytokines in melanoma patients treated with interferon-alpha 2b. Clin Cancer Res 13:2422-2428, 2007

##### Parameter: Autoantibodies and Clinical Manifestations of Autoimmunity

##### Setting: Adjuvant IFN-α Therapy - Melanoma

Gogas H, Ioannovich J, Dafni U, et al.: Prognostic significance of autoimmunity during treatment of melanoma with interferon. *N Engl J Med: 354:709-18, 2006*

##### Parameter: IFN-γ Polymorphism

##### Setting: Immuno (IL-2) Chemotherapy - Metastatic Melanoma

Liu DX, O'Day SJ, Yang DY, et al.: Impact of gene polymorphisms on clinical outcome for stage IV melanoma patients treated with biochemotherapy: An exploratory study. Clin Cancer Res 11:1237-1246, 2005

##### Parameter: IL-6, PPARG

##### Setting: BCG - Bladder Cancer

Leibovici D, Grossman HB, Dinney CP, et al.: Polymorphisms in inflammation genes and bladder cancer: From initiation to recurrence, progression, and survival. J Clin Oncol 23:5746-5756, 2005

#### C. Correlation between Immune Response and Clinical Outcome in Cancer Patients; Positive Studies

Examples

##### Parameter: Melan-A-tetramer+ Immunity

##### Setting: High Dose Poly-Epitope Vaccine - Metastatic Melanoma

Dangoor A, Lorigan P, Keilholz U, et al.: Clinical and immunological responses in metastatic melanoma patients vaccinated with a high-dose poly-epitope vaccine. Cancer Immunol Immunother 59:863-73, 2010

##### Parameter: High Interferon-gamma-associated Proliferative CD4+ T-cell Response and Broad Response of CD8+ Interferon-gamma T Cells

##### Setting: HPV-16 Vaccine Therapy - Vulvar Intraepithelial Neoplasia

Welters MJ, Kenter GG, de Vos van Steenwijk PJ, et al.: Success or failure of vaccination for HPV16-positive vulvar lesions correlates with kinetics and phenotype of induced T-cell responses. Proc Natl Acad Sci U S A. 107:11895-9, 2010

##### Parameter: High Interferon-gamma-associated Proliferative CD4+ T-cell Response and Broad Response of CD8+ Interferon-gamma T Cells

##### Setting: HPV-16 Vaccine Therap - Vulvar Intraepithelial Neoplasia

Kenter GG, Welters MJ, Valentijn AR, et al.: Vaccination against HPV-16 oncoproteins for vulvar intraepithelial neoplasia. N Engl J Med 361:1838-47, 2009

##### Parameter: Tumor Specific Immune Response (ELISPOT)

##### Setting: Tumor Multi-Epitope Vaccine - Metastatic Melanoma

Kirkwood JM, Lee S, Moschos SJ, et al.: Immunogenicity and antitumor effects of vaccination with peptide vaccine+/-granulocyte-monocyte colony-stimulating factor and/or IFN-alpha2b in advanced metastatic melanoma: Eastern Cooperative Oncology Group Phase II Trial E1696. Clin Canc Res 15:1443-1451, 2009

##### Parameter: NY-ESO-1-specific B and T Cell Responses

##### Setting: Anti CTLA-4 Immunotherapy - Metastatic Melanoma

Yuan J, Gnjatic S, Li H, Powel S, et al.: CTLA-4 blockade enhances polyfunctional NY-ESO-1 specific T cell responses in metastatic melanoma patients with clinical benefit. Proc Natl Acad Sci U S A. 105:20410-5, 2008

##### Parameter: Epitope Spreading

##### Setting: Adenovirus MART-1 Dendritic Cell Vaccination - Metastatic Melanoma

Butterfield LH, Comin-Anduix B, Vujanovic L, et al.: Adenovirus MART-1-engineered autologous dendritic cell vaccine for metastatic melanoma. J immunother 31:294-309, 2008

##### Parameter: Epitope Spreading

##### Setting: Dendritic Cell Based Vaccination - Stage II-IV Melanoma

Ribas A, Glaspy JA, Lee Y, et al.: Role of dendritic cell phenotype, determinant spreading, and negative costimulatory blockade in dendritic cell-based melanoma immunotherapy. J Immunother 27:354-367, 2004

##### Parameter: Epitope Spreading

##### Setting: Dendritic Cell Based Vaccination - Stage III-IV Melanoma

Butterfield LH, Ribas A, Dissette VB, et al. Determinant spreading associated with clinical response in dendritic cell-based immunotherapy for malignant melanoma. Clin Canc Res 9:998-1008, 2003

##### Parameter: Epitope Spreading

##### Setting: Peptide-Pulsed Dendritic Cells Vaccination - Metastatic Renal Cell Carcinoma

Wierecky J, Müller MR, Wirths S, et al.: Immunologic and clinical responses after vaccinations with peptide-pulsed dendritic cells in metastatic renal cancer patients. Cancer Res 66:5910-8, 2006

##### Parameter: Epitope Spreading

##### Setting: Her2 Specific Vaccination - Stage III-IV Breast Cancer

Salazar LG, Goodell V, O'Meara M, et al: Persistent immunity and survival after immunization with a HER2/neu (HER2) vaccine. J Clin Oncol 27(15S):2010 (Meeting Abstract)

##### Parameter: Overall Immunity to MelAgs

##### Setting: Dendritic Cell Based Vaccination - Metastatic Melanoma

Banchereau J, Palucka AK, Dhodapkar M, et al. Immune and clinical responses in patients with metastatic melanoma to CD34(+) progenitor-derived dendritic cell vaccine. Cancer Res 6451-8. 2001

##### Parameter: T-cell Response (ELISPOT)

##### Setting: 12-Peptide Vaccine - Stage IIB-IV - Melanoma

Slingluff CL, Jr., Petroni GR, Chianese-Bullock KA, et al.: Immunologic and clinical outcomes of a randomized phase II trial of two multipeptide vaccines for melanoma in the adjuvant setting. Clin Canc Res 13:6386-6395, 2007

##### Parameter: Tumor specific IFN-γ ELISPOT Response

##### Setting: Adjuvant Autologous Tumor Lysate Vaccine - Colon Cancer

Barth RJ Jr, Fisher DA, Wallace PK, et al.: A randomized trial of ex vivo CD40L activation of a dendritic cell vaccine in colorectal cancer patients: tumor-specific immune responses are associated with improved survival. Clin Cancer Res 16:5548-56, 2010

##### Parameter: Delayed Type IV Hypersensitivity

##### Setting: Dendritic Cell Vaccine - Stage III-IV Melanoma Patients

López MN, Pereda C, Segal G, et al: Prolonged survival of dendritic cell-vaccinated melanoma patients correlates with tumor-specific delayed type IV hypersensitivity response and reduction of tumor growth factor beta-expressing T cells. J Clin Oncol 27:945-52, 2009

##### Parameter: Delayed Type IV Hypersensitivity

##### Setting: Adjuvant Autologous Tumor Cell Vaccination - Stage III-IV Melanoma Patients

Baars A, Claessen AM, van den Eertwegh AJ et al.: Skin tests predict survival after autologous tumor cell vaccination in metastatic melanoma: experience in 81 patients. Ann Oncol 11:965-70, 2000

##### Parameter: Delayed Type IV Hypersensitivity

##### Setting: Allogeneic GM-CSF - Stage I-III Pancreatic Cancer

Jaffee EM, Hruban RH, Biedrzycki B, et al.: Novel allogeneic granulocyte-macrophage colony-stimulating factor-secreting tumor vaccine for pancreatic cancer: a phase I trial of safety and immune activation. J Clin Oncol 19: 45-56, 2009

##### Parameter: Delayed Type IV Hypersensitivity

##### Setting: Dendritic Cell Vaccination - Stage IV Melanoma

de Vries IJ, Bernsen MR, Lesterhuis WJ, et al: Immunomonitoring tumor-specific T cells in delayed-type hypersensitivity skin biopsies after dendritic cell vaccination correlates with clinical outcome. J Clin Oncol 23:5779-87, 2005

### 3. Gene Expression Profiling in Cancer Immunotherapy

Examples

Gajewski TF, Fuertes M, Spaapen R et al.: Molecular profiling to identify relevant immune resistance mechanisms in the tumor microenvironment. Curr Opin Immunol 23:286-92, 2011

Bedognetti D, Wang E, Sertoli MR, Marincola FM: Gene-expression profiling in vaccine therapy and immunotherapy for cancer. Expert Rev Vaccines 6:555-65, 2010

Worschech A, Chen NH, Yu YA, et al.: Systemic treatment of xenografts with vaccinia virus GLV-1h68 reveals the immunologic facet of oncolytic therapy. BMC Genomics 10, 2009

Harlin H, Meng Y, Peterson AC, et al.: Chemokine expression in melanoma metastases associated with CD8(+) T-cell recruitment. Cancer Res 69:3077-3085, 2009

Wang E, Miller LD, Ohnmacht GA, et al.: Prospective molecular profiling of melanoma metastases suggests classifiers of immune responsiveness. Cancer Res 62:3581-3586, 2002

Panelli MC, Stashower ME, Slade HB, et al.: Sequential gene profiling of basal cell carcinomas treated with imiquimod in a placebo-controlled study defines the requirements for tissue rejection. Genome Biol 8:R8, 2007

Sullivan RJ, Hoshida Y, Brunet J, et al.: A single center experience with high-dose (HD) IL-2 treatment for patients with advanced melanoma and pilot investigation of a novel gene expression signature as a predictor of response. J Clin Oncol 27:9003, 2009 (Meeting Abstract)

Gajewski TF, Zha Y, Thurner B, et al.: Association of gene expression profile in metastatic melanoma and survival to a dendritic cell-based vaccine. J Clin Oncol 27:9002, 2009 (Meeting Abstract)

Louahed J, Gruselle O, Gaulis S, et al.: Expression of defined genes identified by pretreatment tumor profiling: Association with clinical responses to the GSK MAGE- A3 immunotherapeutic in metastatic melanoma patients (EORTC 16032-18031). J Clin Oncol 26:9045, 2008 (Meeting Abstract)

Vansteenkiste JF, Zielinski, M, Dahabreh IJ, et al.: Association of gene expression signature and clinical efficacy of MAGE-A3 antigen-specific cancer immunotherapeutic (ASCI) as adjuvant therapy in resected stage IB/II non-small cell lung cancer (NSCLC). J Clin Oncol 26:7501, 2008 (Meeting Abstract)

Mandruzzato S, Callegaro A, Turcatel G, et al.: A gene expression signature associated with survival in metastatic melanoma. J Transl Med. 4:50, 2006

Panelli MC, Wang E, Phan G, et al.: Gene-expression profiling of the response of peripheral blood mononuclear cells and melanoma metastases to systemic IL-2 administration. Genome Biol 3, 2002

### 4. Useful Reviews

Marincola FM: The trouble with translational medicine. J Intern Med. 2011 May

16. [Epub ahead of print]

Kirkwood JM, Butterfield LH, Tarhini AA, Zarour H, Kalinski P, Ferrone S: Immunotherapy of Cancer in 2011 CA: A Cancer Journal for Clinicians [in press]

Ascierto ML, De Giorgi V, Liu Q et al.: An immunologic portrait of cancer. Journal of Translational Medicine, (2011, in press).

Gajewski TF, Fuertes M, Spaapen R, et al.: Molecular profiling to identify relevant immune resistance mechanisms in the tumor microenvironment. Curr Opin Immunol 23:286-92, 2011

Disis ML: Immunologic biomarkers as correlates of clinical response to cancer immunotherapy. Cancer Immunol Immunother. 60:433-42, 2011

Sznol M: Molecular markers of response to treatment for melanoma Cancer J 17:127-33, 2011

Bedognetti D, Wang E, Sertoli MR, Marincola FM. Gene-expression profiling in vaccine therapy and immunotherapy for cancer. Expert Rev Vaccines 6:555-65, 2010

Slota M, Lim JB, Dang Y, Disis ML: ELISpot for measuring human immune responses to vaccines. Expert Rev Vaccines 10:299-306, 2011

Gogas H, Eggermont AM, Hauschild et al: Biomarkers in melanoma. Ann Oncol. 20 Suppl 6:vi8-13, 2009

Simon R: Translational research in oncology: key bottlenecks and new paradigms. Expert Rev Mol Med 12:e32, 2010

Kirkwood JM, Tarhini AA, Panelli MC, Moschos SJ, Zarour HM, Butterfield LH,

Gogas HJ: Next generation of immunotherapy for melanoma. J Clin Oncol 26:3445-55, 2008

Freidlin B, McShane LM, Korn EL: Randomized clinical trials with biomarkers:

design issues. J Natl Cancer Inst 3;102:152-60, 2011

### 5. Bioinformatics for Biomarkers Discovery

Zhao Y, Simon R: Development and validation of predictive indices for a continuous outcome using gene expression profiles. Cancer Inform 9:105-114, 2010

Chaussabel D: Data management: it starts at the bench. Nat Immunol. 10:1225-7, 2009

Chaussabel D, Quinn C, Shen J, et al.: A modular analysis framework for blood genomics studies: Application to systemic lupus erythematosus. Immunity 29:150-164, 2008

Bredel M, Scholtens D, Harsh GR, et al.: Model of a cooperative genetic landscape in gliomas. Neuro-Oncology 11:606, 2009

Simon R: The use of genomics in clinical trial design. Clin Cancer Res 14:5984-5993, 2008

Simon R: Development and validation of biomarker classifiers for treatment selection. J Stat Plan Infer 138:308-320, 2008

Nacu S, Critchley-Thorne R, Lee P, et al.: Gene expression network analysis and applications to immunology. Bioinformatics 23:850-858, 2007

Dupuy A, Simon RM: Critical review of published microarray studies for cancer outcome and guidelines on statistical analysis and reporting. J Natl Cancer Inst 99:147-57, 2007

### 6. New Technologies for Biomarkers Discovery

Linnarsson S: Recent advances in DNA sequencing methods - general principles of sample preparation. Exp Cell Res 316:1339-1343, 2010

Metzker ML: Sequencing technologies - the next generation. Nat Rev Genet 11:31-46, 2010

Maecker HT, Nolan GP, Fathman CG: New technologies for autoimmune disease monitoring. Curr Opin Endocrinol Diabetes Obes 17:322-328, 2010

Ornatsky O, Bandura D, Baranov V, et al.: Highly multiparametric analysis by mass cytometry. J Immunol Method 361:1-20, 2010

Newell EW, Klein LO, Yu W, Davis MM.: Simultaneous detection of many T-cell specificities using combinatorial tetramer staining. Nat Methods 6:497-9, 2009

Hadrup SR, Bakker AH, Shu CJ, et al.: Parallel detection of antigen-specific T-cell responses by multidimensional encoding of MHC multimers. Nat Methods 6:520-6, 2009

Lugli E, Roederer M, Cossarizza A: Data analysis in flow cytometry: the future just started. Cytometry A 77:705-713, 2010

Ryan BM, Robles AI, Harris CC: Genetic variation in microRNA networks: the implications for cancer research. Nat Rev Cancer 10:389-402, 2010

Collas P: The current state of chromatin immunoprecipitation. Mol Biotechnol 45:87-100, 2010

Espina V, Wulfkuhle J, Liotta LA: Application of laser microdissection and reverse-phase protein microarrays to the molecular profiling of cancer signal pathway networks in the tissue microenvironment. Clin Lab Med 29:1-13, 2009

Luchini A, Longo C, Espina V, et al.: Nanoparticle technology: Addressing the fundamental roadblocks to protein biomarker discovery. J Mater Chem 19:5071-5077, 2009

Park PJ: ChIP-seq: advantages and challenges of a maturing technology. Nat Rev Genet 10:669-680, 2009

Hanash SM, Pitteri SJ, Faca VM: Mining the plasma proteome for cancer biomarkers. Nature 452:571-579, 2008

Wang E, Marincola FM: Bottom up: a modular view of immunology. Immunity 2:9-11, 2008

Gulmann C, Sheehan KM, Kay EW, et al.: Array-based proteomics: mapping of protein circuitries for diagnostics, prognostics, and therapy guidance in cancer. J Pathol 208:595-606, 2006

DeAngelis JT, Farrington WJ, Tollefsbol TO: An overview of epigenetic assays. Mol Biotechnol 38:179-183, 2008

Gilad Y, Borevitz J: Using DNA microarrays to study natural variation. Curr Opin Genet Dev 16:553-558, 2006

Speer R, Wulfkuhle JD, Liotta LA, et al.: Reverse-phase protein microarrays for tissue-based analysis. Curr Opin Mol Ther 7:240-245, 2005

Perfetto SP, Chattopadhyay PK, Roederer M: Seventeen-colour flow cytometry: unravelling the immune system. Nat Rev Immunol 4:648-655, 2004

Hanash S: Disease proteomics. Nature 422:226-232, 2003

### 7. Standardization and Harmonization of Sample Collection and Use of Immunological Assays

#### A. Processing, Storage and Shipping of Blood Samples and Serum for Immunological Studies - Technical Considerations

Examples

Disis ML, dela Rosa C, Goodell V, et al.: Maximizing the retention of antigen specific lymphocyte function after cryopreservation. J Immunol Methods 308:13-18, 2006

Bull M, Lee D, Stucky J, et al.: Defining blood processing parameters for optimal detection of cryopreserved antigen-specific responses for HIV vaccine trials. J Immunol Methods 322:57-69, 2007

Kierstead LS, Dubey S, Meyer B, et al.: Enhanced rates and magnitude of immune responses detected against an HIV vaccine: effect of using an optimized process for isolating PBMC. AIDS Res Human Retroviruses 23:86-92, 2007

Ruitenberg JJ, Mulder CB, Maino VC, et al.: VACUTAINER CPT and Ficoll density gradient separation perform equivalently in maintaining the quality and function of PBMC from HIV seropositive blood samples. BMC Immunol 7:11, 2006

Tree TI, Roep BO, Peakman M.: Enhancing the sensitivity of assays to detect T cell reactivity: the effect of cell separation and cryopreservation media. Ann N Y Acad Sci 1037:26-32, 2004

Smith JG, Joseph HR, Green T, et al.: Establishing acceptance criteria for cell-mediated immunity assays using frozen peripheral blood mononuclear cells stored under optimal and suboptimal conditions. Clin Vaccine Immunol 14:527-37, 2007

McKenna KC, Beatty KM, Vicetti Miguel R, Bilonick RA: Delayed processing of blood increases the frequency of activated CD11b+ CD15+ granulocytes which inhibit T cell function. J Immunol Method 341:68-75, 2009

#### B. Cellular Immunotherapy: Characterization of Cellular Products

Examples

Jin P, Han TH, Ren J, Saunders S, et al.: Molecular signatures of maturing dendritic cells: implications for testing the quality of dendritic cell therapies. J Transl Med 8:4, 2010

Sheikh NA, Jones LA: CD54 is a surrogate marker of antigen presenting cell activation. Immunol Immunother 57:1381-1390, 2008

Higano CS, Schellhammer PF, Small EJ, et al.: Integrated data from 2 randomized, double-blind, placebo-controlled, phase 3 trials of active cellular immunotherapy with Sipuleucel-T in advanced prostate cancer. Cancer 115:3670-3679, 2009

Butterfield LH, Gooding W, Whiteside TL: Development of a potency assay for human dendritic cells: IL-12p70 production. J Immunother 31:89-100, 2008

Ayache S, Panelli M, Marincola FM, et al.: Effects of storage time and exogenous protease inhibitors on plasma protein levels. American Journal of Clinical Pathology 126:174-184, 2006

#### C. Assay Standardization and Harmonization

Examples

Clinical Laboratory Improvement Amendments (CLIA) Guideline: http://www.cms.hhs.gov/CLIA/:

International Conference on Harmonization of Technical Requirements for Registration of Pharmaceuticals for Human Use (ICH) - common platform of Europe, Japan and United States authorities: http://www.ich.org/products/guidelines.html

Attig S, Price L, Janetzki S, Kalos M, Pride M, McNeil L, Clay T, Yuan J, Odunsi K, Hoos A, Romero P, Britten CM, Assay Working Group CC. A critical assessment for the value of markers to gate-out undesired events in HLA-peptide multimer staining protocols. J Transl Med, 9:108, 2011

Maecker HT, Hassler J, Payne JK, et al.: Precision and linearity targets for validation of an IFNgamma ELISPOT, cytokine flow cytometry, and tetramer assay using CMV peptides. BMC Immunol 9:9, 2008

Nomura L, Maino VC, Maecker HT: Standardization and optimization of multiparameter intracellular cytokine staining. Cytometry A 73:984-991, 2008

Afonso G, Scotto M, Renand A, et al.: Critical parameters in blood processing for T-cell assays: validation on ELISpot and tetramer platforms. J Immunol Methods 359:28-36, 2010

Britten CM, Gouttefangeas C, Welters MJ, et al.: The CIMT-monitoring panel: a two-step approach to harmonize the enumeration of antigen-specific CD8+ T lymphocytes by structural and functional assays. Cancer Immunol Immunother 57:289-302, 2008

Britten CM, Janetzki S, Ben Porat L, et al.: Harmonization guidelines for HLA-peptide multimer assays derived from results of a large scale international proficiency panel of the Cancer Vaccine Consortium. Cancer Immunol Immunother 2009:1701-1713, 2009

Britten CM, Janetzki S, van der Burg SH, et al.: Toward the harmonization of immune monitoring in clinical trials: quo vadis? Cancer Immunol Immunother 57:285-288, 2008

Mander A, Gouttefangeas C, Ottensmeier C, et al.: Serum is not required for ex vivo IFN-gamma ELISPOT: a collaborative study of different protocols from the European CIMT Immunoguiding Program. Cancer Immunol Immunother 59:619-627

Fahey JL, Aziz N, Spritzler J, et al.: Need for an external proficiency testing program for cytokines, chemokines, and plasma markers of immune activation. Clin Diagn Lab Immunol 7:540-548, 2000

Denny TN, Gelman R, Bergeron M, et al.: A North American multilaboratory study of CD4 counts using flow cytometric panLeukogating (PLG): a NIAID-DAIDS Immunology Quality Assessment Program Study. Cytometry B Clin Cytom 74 Suppl 1: S52-S64, 2008

Boaz MJ, Hayes P, Tarragona T, et al.: Concordant proficiency in measurement of T-cell immunity in human immunodeficiency virus vaccine clinical trials by peripheral blood mononuclear cell and enzyme-linked immunospot assays in laboratories from three continents. Clin Vaccine Immunol 16:147-155, 2009

Smith SG, Joosten SA, Verscheure V, et al.: Identification of major factors influencing ELISpot-based monitoring of cellular responses to antigens from Mycobacterium tuberculosis. PLoS One 4:e7972, 2009

Hanekom WA, Dockrell HM, Ottenhoff TH, et al.: Immunological outcomes of new tuberculosis vaccine trials: WHO panel recommendations. PLoS Med 5:e145, 2008

Schloot NC, Meierhoff G, Karlsson Faresjö M, et al.: Comparison of cytokine ELISpot assay formats for the detection of islet antigen autoreactive T cells. Report of the third immunology of diabetes society T-cell workshop. J Autoimmun 21:365-76, 2003

Moodie Z, Price L, Gouttefangeas C, et al.: Response definition criteria for ELISPOT assays revisited. Cancer Immunol Immunother 59:1489-501, 2010

#### D. Assays for Determination of Antitumor Immune-Response in Clinical Trials

Examples

##### ELISPOT

Quast S, Zhang W, Shive C, et al.: IL-2 absorption affects IFN-gamma and IL-5, but not IL-4 producing memory T cells in double color cytokine ELISPOT assays. Cell Immunol 237:28-36, 2005

##### Granzyme B ELISPOT

Shafer-Weaver K, Rosenberg S, Strobl S, et al.: Application of the granzyme B ELISPOT assay for monitoring cancer vaccine trials. J Immunother 29:328-335, 2006

##### Modified ELISPOT

Malyguine A, Strobl SL, Shafer-Weaver KA, et al.: A modified human ELISPOT assay to detect specific responses to primary tumor cell targets. J Trans Med 2:9, 2004

##### FACS-based Cytotoxicity Assay

Kim GG, Donnenberg VS, Donnenberg AD, et al.: A novel multiparametric flow cytometry-based cytotoxicity assay simultaneously immunophenotypes effector cells: comparisons to a 4 h 51Cr-release assay. J Immunol Methods 325:51-66, 2007

Devevre E, Romero P, Mahnke YD: LiveCount Assay: concomitant measurement of cytolytic activity and phenotypic characterisation of CD8(+) T-cells by flow cytometry. J Immunol Methods 311:31-46, 2006

Zaritskaya L, Shafer-Weaver KA, Gregory MK, et al.: Application of a flow cytometric cytotoxicity assay for monitoring cancer vaccine trials. J Immunother 32:186-194, 2009

Maecker HT, Dunn HS, Suni MA, et al.: Use of overlapping peptide mixtures as antigens for cytokine flow cytometry. J Immunol Methods 255:27-40, 2001

##### FACS/ELISPOT/TETRAMERS

Whiteside TL, Zhao Y, Tsukishiro T, Elder EM, Gooding W, Baar J: Enzyme-linked immunospot, cytokine flow cytometry, and tetramers in the detection of T-cell responses to a dendritic cell-based multipeptide vaccine in patients with melanoma. Clin Cancer Res, 2003

### 8. Reporting Biomarkers Data in Publications

Examples

#### REMARK Recommendations

(accepted by most major journal included those published by the American Society of Clinical Oncology and the American Association for Cancer Research)

McShane LM, Altman DG, Sauerbrei W, et al.: Reporting recommendations for tumor marker prognostic studies (REMARK). J Natl Canc Inst 97:1180-1184, 2005

#### MIBBI Project - Guidelines Promotion

http://www.mibbi.org

Taylor CF, Field D, Sansone SA, et al.: Promoting coherent minimum reporting guidelines for biological and biomedical investigations: the MIBBI project. Nat Biotechn 26:889-896, 2008

#### MIAME - Microarray Experiment

Brazma A, Hingamp P, Quackenbush J, et al.: Minimum information about a microarray experiment (MIAME)-toward standards for microarray data. Nat Genet 29:365-371, 2001

#### MIATA - T Cell Assays

http://www.miataproject.org

Janetzki S, Britten CM, Kalos M, et al.: "MIATA"-minimal information about T cell assays. Immunity 31:527-528, 2009

Britten CM, Janetzki S, van der Burg SH, et al: Minimal information about T cell assays: the process of reaching the community of T cell immunologists in cancer and beyond. Cancer Immunol Immunother 60:15-22, 2011

#### MIFlowCyt - Flow Cytometry

Lee JA, Spidlen J, Boyce K, et al.: MIFlowCyt: the minimum information about a Flow Cytometry Experiment. Cytometry A 73:926-930, 2008

#### Data File for Flow Cytometry

Spidlen J, Moore W, Parks D, et al.: Data file standard for flow cytometry, version FCS 3.1. Cytometry A 77:97-100, 2010

## Part II. High Throughput and New Technologies for Biomarker Discovery: Arrays, Platforms, Tools for The Bench and Online Resources

### 1. Genomic Biomarkers Discovery

#### A. Single Nucleotide Polymorphisms (SNPs) Arrays

Examples

Name: Affymetrix Genome-Wide Human SNP Array 6.0

*Comment:*1.8 million genetic markers, including more than 906,600 single nucleotide polymorphisms (SNPs) and more than 946,000 probes for the detection of copy number variation

*Website:*http://www.affymetrix.com/browse/products.jsp?productId=131533&navMode=34000&navAction=jump&aId=productsNav#1_1

Name: Affymetrix DMET Plus Premier Pack

*Comment:*Drug metabolism studies. Coverage of a wide range of genetic variations, including common and rare SNPs, insertions, deletions, tri-alleles, and copy number. 1,936 drug metabolism markers in 225 genes

*Website:*http://www.affymetrix.com/browse/products.jsp?productId=131412&navMode=34000&navAction=jump&aId=productsNav#1_1

Name: Illumina Omni Microarray

*Comment:*Next generation genome-wide association studies. The Omni family of microarrays will soon allow researchers to assay up to 5 million markers per sample, including comprehensive coverage of both common and rare variants identified by 1000 Genomes Project. As novel SNP sets are released into the public database, researchers using Omni products will have exclusive access to supplemental arrays that build up to the full 5 million variants

*Website:*http://www.illumina.com/landing/click/gwas.ilmn?scid=2011110PPC1&gclid=CJij6ffe26kCFQPc4AodcmxEZQ

Name: Fluidigm Dynamic Array for SNP Genotyping

*Comment:*The Fluidigm Dynamic Arrays allow you to use your existing TaqMan^® ^SNP Genotyping assays in a flexible and cost effective fashion. Each dynamic array allows you to setup up to 9,216 individual TaqMan reactions in a single experiment

*Website:*http://www.expressionanalysis.com/services/category/fluidigm_dna_focused_set_snp

Name: TaqMan^® ^Pre-Designed SNP Genotyping Assays

*Comment:*Includes over 4.5 million SNP assays, including 3.5 million HapMap, and ~70,000 coding SNP assays. This collection now includes ~160,000 validated assays with associated minor allele frequency data available

*Website:*https://products.appliedbiosystems.com/ab/en/US/adirect/ab?cmd=catNavigate2&catID=600769&tab=TechSpec

#### B. Comparative Genomic Hybridization (CGH) Arrays

Examples

Name:*Agilent Human Genome CGH Microarrays*

Comment: High-resolution tool for genome-wide DNA copy number variation profiling. Comprehensive probe coverage is enhanced with emphasis on known genes, promoters, miRNAs, pseudoautosomal, and telomeric regions

Website:http://www.genomics.agilent.com/CollectionSubpage.aspx?PageType=Product&SubPageType=ProductDetail&PageID=1463

Name: Nimblegen Cytogenetics (CGX) Arrays

*Comment: *Genome-wide analysis of DNA copy number changes with a subset of probes focused in disease-associated regions

*Website: *http://www.nimblegen.com/products/cgh/human.html#cyto

#### C. Mitochondrial Genome Arrays

Example

Name: Affymetrix GeneChip^® ^Human Mitochondrial Resequencing Array 2.0

*Comment:*Detection of germ line and heteroplasmic mutations by delivering the complete mitochondrial genome with minimal PCR in only 48 hours

*Website:*http://www.affymetrix.com/support/technical/byproduct.affx?product=humitoreseq

### 2. Epigenomic Biomarkers Discovery

#### A. Methylation Arrays

Examples

Name: Agilent Human DNA Methylation Microarrays

*Comment:*The array is specifically designed for analysis of methylated DNA derived from affinity-based isolation methods such as methylated DNA immunoprecipitation (MDIP)

*Website:*http://www.genomics.agilent.com/CollectionSubpage.aspx?PageType=Product&SubPageType=ProductDetail&PageID=2157

Name: Nimblegen 2.1 M Whole-Genome Tiling Set

*Comment:*Whole-genome formats are available in two versions: a 10-array set at 100 bp probe interval or a 4-array set at > 200 bp probe interval

*Website:*http://www.nimblegen.com/products/methylation/whole_genome.html

Name: Illumina 450 K Infinium Methylation BeadChip Kit

*Comment:*This assay allow to interrogate > 450,000 methylation sites per sample at single-nucleotide resolution

*Website:*http://www.illumina.com/products/methylation_450_beadchip_kits.ilmn

#### B. microRNA (miRNA) Arrays

Examples

Name: Illumina MicroRNA Universal Array Matrix

*Comment:*miRNA analysis

*Website:*http://www.illumina.com/products/microrna_universal_array_matrix.ilmn

Name: Affymetrix GeneChip miRNA Array

*Comment:*miRNA analysis

*Website:*http://www.affymetrix.com/estore/browse/products.jsp;jsessionid=7386716AB0BD679701B518044ADA58F5?navMode=34000&productId=131473&navAction=jump&aId=productsNav

Name: Exiqon's miRCURY LNA microRNA Array

*Comment:*miRNA analysis

*Website:*http://www.exiqon.com/microrna-microarray-analysis

#### C. Chromatin Immunoprecipitation (ChIP) Arrays (ChIP on chip)

[ChIP-on-chip (chromatin immunoprecipitation-on-chip), also known as Location Analysis (LA), is a high throughput (genome-wide) identification and analysis of DNA fragments that are bound by specific proteins such as histones, transcriptional factors and polymerases]

Examples

Name: Nimblegen 2.1 M ChIP-Chip Array

*Comment:*ChIP-chip analysis

*Website:*http://www.nimblegen.com/products/chip/index.html

Name: Agilent SurePrint G3 Human Promoter Microarray

*Comment:*ChIP-chip analysis

*Website:*http://www.genomics.agilent.com/CollectionSubpage.aspx?PageType=Product&SubPageType=ProductDetail&PageID=1487

Name: Affymetrix GeneChip Tiling Arrays

*Comment:*ChIP-chip analysis

*Website:*http://www.affymetrix.com/estore/browse/level_three_category_and_children.jsp?parent=35808&expand=true&category=35818&fromAccordionMenu=true&subCategory=35818

### 3. Transcriptomic Biomarkers Discovery

#### A. Expression Arrays

Examples

Name: Affymetrix Gene Chip Human Exon 1.0 ST Array

*Comment:*Whole genome microarray. With approximately four probes per exon and roughly 40 probes per gene, the GeneChip Human Exon 1.0 ST Array enables two complementary levels of analysis--gene expression and alternative splicing

*Website:*http://www.affymetrix.com/estore/browse/products.jsp?navMode=34000&productId=131453&navAction=jump&aId=productsNav#1_1

Name: Agilent SurePrint G3 Human GE 8 × 60 K Kit

*Comment:*Whole genome microarray. The SurePrint G3 Human GE 8 × 60 K Microarrays and Human GE 4 × 44 K v2 Microarrays are based on updated transcriptome databases for mRNA targets, while the SurePrint G3 arrays also include probes for lincRNAs (long intergenic non-coding RNAs). With the combination of mRNA and lincRNAs, it is possible to perform two experiments on a single microarray, confidently predicting lincRNA function

*Website:*http://www.genomics.agilent.com/CollectionSubpage.aspx?PageType=Product&SubPageType=ProductDetail&PageID=1515

Name: Illumina HumanHT-12 v4 Expression BeadChip Kits

*Comment:*The HumanHT-12 v4 Expression BeadChip provides high throughput processing of 12 samples per BeadChip. Each array on the HumanHT-12 v4 Expression BeadChip targets more than 31,000 annotated genes with more than 47,000 probes

*Website:*http://www.illumina.com/products/humanht_12_expression_beadchip_kits_v4.ilmn

#### B. Quantitative Assays

Examples

Name: QuantiGene^® ^Plex 2.0 Reagent System (Luminex Assay)

*Comment:*Quantitatively measure 3-36 RNA targets simultaneously

*Website:*http://www.panomics.com/index.php?id=product_6

Name: Procarta Transcription Factor Profiling Kits (Luminex Assay)

*Comment:*Quantitate the DNA binding activity of up to 44 different transcription factors (TFs) in a single well using nuclear extracts or whole cell lysates

*Website:*http://www.panomics.com/index.php?id=product_17

Name: TaqMan^® ^Probe-Based Gene Expression Analysis

*Comment:*Quantitatively measure 1-384 RNA targets simultaneously

*Website:*http://www.appliedbiosystems.com/absite/us/en/home/applications-technologies/qpcr-real-time-pcr/taqman-probe-based-gene-expression-analysis.html

Name: Fluidigm Dynamic Array for Single Cell Gene Expression

*Comment:*The Fluidigm Dynamic Array enables you to test up to 96 individual cells against 96 genes in a single experiment. The dynamic array assembles the cDNA material from individual cells and reagents to create individual qPCR reactions

*Website:*http://www.fluidigm.com/docs/Application_Note_Dynamic_Array_for_Single-Cell_GE_Analysis.pdf

Name: Nanostring nCounter Analysis System for digital gene expression analysis

*Comment: *The nCounter Analysis System utilizes a novel digital technology that is based on direct multiplexed measurement of gene expression and offers high levels of precision and sensitivity (< 1 copy per cell). The technology uses molecular "barcodes" and single molecule imaging to detect and count hundreds of unique transcripts in a single reaction.

*Website: *http://www.nanostring.com/applications/technology/

### 4. Proteomic Biomarkers Discovery

#### A. Protein and Phosphoprotein Assays

Examples

Name: Nodality

*Comment:*Single-cells network profiling (SCNP): advanced multiparametric quantitative flow cytometry to measure compound effects on multiple signaling cascades in a cell-type-specific manner

*Website:*http://www.nodalityinc.com/

Name: NanoPro Immunoassay

*Comment:*Characterization of proteins in extremely small and precious samples. Unlike traditional protein analysis techniques which require thousands to millions of cells, these assays require as few as 25 cells per assay

*Website:*http://www.cellbiosciences.com/nanopro.html

Name: Invitrogen Immunoassays

*Comment:*Wide range of immunoassays using antibody pairs for single-analyte (ELISA kits) as well as multi-analyte (Lumibex assays) analysis or accurate quantitation of intracellular or extracellular proteins

*Website:*http://www.invitrogen.com/site/us/en/home/Products-and-Services/Applications/Cell-and-Tissue-Analysis/Immunoassays.html

Name: Procarta Cytokine Profiling Assays (Luminex Assay)

*Comment:*Quantitatively measure 3-30 cytokine proteins simultaneously from a variety of matrices including cell culture supernatants, serum or plasma

*Website:*http://www.panomics.com/index.php?id=products_luminexAssays

Name: SearchLight Multiplex Immunoassay Kits

*Comment:*As many as 16 proteins (4 × 4 array in each well) can be measured per well simultaneously with each 50 μl sample

*Website:*http://www.aushon.com/Products-and-Services.php

Name: MSD Multiplex Cytokines and Chemokines

*Comment:*96-well; multiplex cytokine/chemokine kits for up to ten (10) analytes per well.

384-well: Order multiplex cytokine/chemokine kits for up to four (4) analytes per well

*Website:*http://www.mesoscale.com

Name: Zeptomark

*Comment:*Protein profiling for analysis of cell signaling pathways

*Website:*http://www.zeptosens.com/en/

Name: Signaling Protein Luminex Assays

*Comment:*Multiplex assays for measuring signal transduction, post-translational modification & cleaved proteins in multiple research areas, using Luminexbead-based technology

*Website:*http://www.invitrogen.com/site/us/en/home/Products-and-Services/Applications/Cell-and-Tissue-Analysis/Immunoassays/Immunoassays-misc/Multiplex-Bead-Based-Luminex-Technology/Luminex-Assays.html

Name: VeraCode Carboxyl Bead Sets and BeadXpress Reader

Comment:Multiplexed protein arrays (up to 48 immunoassays in a single reaction in a standard 96-well microplate: detection of protein as low as 10 pg/ml)

Website:http://www.illumina.com/products/veracode_carboxyl_bead_sets.ilmn

Name: Procarta SH2 Domain Plex Profiling Kits (Luminex Assay)

*Comment:*Profile phosphotyrosine protein interactions against 30 SH2 binding in a single well using treated and untreated cell lysates. Protein-Protein interaction

*Website:*http://www.panomics.com/downloads/13_4_SH2UM_2_V1.pdf

Name: Proto-Array

*Comment:*Advanced, high-content, functional protein microarray enables to scan thousands of proteins (> 9000) in as little as one day. Highly sensitive results for protein-protein interaction, kinase substrate identification, and serum profiling studies

*Website:*http://www.invitrogen.com/protoarray

Name: Metal Nanoparticle Probes and Dynamic Light Scattering

*Comment:*This assay is based on the use of gold nanoparticle probes combined with dynamic light scattering (DLS) technique, named nanoDLSAY, a highly sensitive, fast and convenient one-step homogeneous immunoassay for monitoring and detecting biotargets, including cancer biomarkers

*Website:*http://tt.research.ucf.edu/LinkClick.aspx?fileticket=6p5A2nGSYzA%3d&tabid=119

#### B. Multicolor Cytometric Systems

Examples

Name: BD LSRFortessa™ Cell Analyzer

*Comment:*Up to 4 lasers to detect up to 18 colors simultaneously

*Website:*http://www.bdbiosciences.com/instruments/lsr/index.jsp

Name: The MACSQuant^® ^Analyzer

*Comment: Three lasers, up to 8 colors*, compact benchtop flow cytometer that is small in size; *MACSQuant^® ^Analyzer *allows researchers to perform sensitive rare cell analysis using magnetic separation

*Website:*http://www.miltenyibiotec.com/downloads/6760/6764/18602/MQ_brochure.pdf

Name: CyAn ADP Analyzer

*Comment:*Up to 11 standard parameters and 9 colors

*Website:*http://www.coulterflow.com/bciflow/instrumentsus.php

Name: Partec CyFlow^® ^ML

*Comment:*3-laser configuration to detect up to 16 colors

*Website:*http://www.partec.com/cms/front_content.php?idcat=13

Name: Accuri C6

*Comment:*Standard 2-laser configuration, 4- color system

*Website:*http://www.accuricytometers.com/products/

### 5. Next Generation Sequencing: Whole Genomic and Transcriptomic Sequencing Systems

Next generation sequencing, possible applications: Whole Genome and Transcriptome Sequencing-Based analysis, Gene Regulation Analysis, SNP Discovery and Structural Variation Analysis, Cytogenetic Analysis, DNA-Protein Interaction Analysis (ChIP-Seq), Sequencing-Based Methylation Analysis, Small RNA Discovery and Analysis

Examples

*Name: Illumina *- several systems, examples: HiSeq 2000 (Sequencing); HiScanSQ (Sequencing + Arrays) and MiSeq

*Comment:*Next generation sequencing

*Website:*http://www.illumina.com/systems.ilmn

*Name: Roche *- several systems, examples: Genome Sequencer FLX System and GS Junior System (454 Sequencing)

*Comment:*Next generation sequencing

*Website:*http://www.454.com/

*Name: Applied Biosystems *- several systems, examples: Solid 4 System and Solid PI System (Solid System)

*Comment:*Next generation sequencing

*Website:*http://www.appliedbiosystems.com/absite/us/en/home/applications-technologies/solid-next-generation-sequencing.html

Name: Ion Torrent PGM

*Comment:*Next generation sequencing: semiconductor technology allows runs in about 2 hours.

*Website:*http://www.iontorrent.com/products-ion-pgm/

*Name:*PacBio *RS*

*Comment:*Third generation sequencing: Long readlengths, single molecule sequencing

*Website:*http://www.pacificbiosciences.com/products

### 6. Software and Tools for Data Analysis

#### A. Microarrays/Sequencing Data Analysis

Examples:

Name: BRB - ArrayTools

*Comment:*Microarray/array CGH analysis. BRB-Array Tools was developed by Dr. Richard Simon and BRB-ArrayTools Development Team, NCI, NIH. The program can be used for non-commercial purposes free-of-charge

*Website:*http://linus.nci.nih.gov/pilot/index.htm

*License needed:*No

Name: Partek

*Comment:*Next generation sequencing technologies, including gene expression and DGE, RNA-seq and alternative splicing, copy number and association, ChIP-chip, ChIP-seq, microRNA and SNP association study

*Website:*http://www.partek.com/

*License needed:*Yes

Name: Nexus Copy Number

*Comment:*aCGH and SNP copy number analysis

*Website:*http://www.biodiscovery.com/index/nexus

*License needed:*Yes

Name: Nexus Expression

*Comment:*Microarray gene expression analysis

*Website:*http://www.biodiscovery.com/index/nexus-expression

*License needed:*Yes

*Name: mAdb *(aka *Mad Bee)*

*Comment:*Microarray gene expression analysis

*Website:*http://madb.nci.nih.gov/

*License needed:*No

Name: Bioconductor

*Comment:*Tools for the analysis and comprehension of high-throughput genomic data. Bioconductor uses the R statistical programming language

*Website:*http://www.bioconductor.org/

*License needed:*No

Name: MATLAB 7.11

*Comment:*MATLAB is a high-level technical computing language and interactive environment for algorithm development, data visualization, data analysis, and numeric computation

*Website:*http://www.mathworks.com/products/matlab/description1.html

*License needed:*Yes

Name: GeneSifter^® ^Analysis

*Comment:*Software microarray and next generation sequencing analysis

*Website:*http://www.geospiza.com/Contact/genesiftertrial_ng.shtml

*License needed:*Yes

Name: ADaCGH

*Comment:*Web tool for the analysis of aCGH data sets

*Website:*http://adacgh.bioinfo.cnio.es/

*License needed:*No

Name: ArrayStar

*Comment:*Gene expression analysis software package that includes visualization tools to help analyze microarray data, including Venn diagrams, a scatter plot, heat maps and line graphs for clustering, and a gene ontology tree

*Website:*http://www.dnastar.com/t-products-arraystar.aspx

*License needed:*Yes

Name: GeneSpring GX

*Comment:*Statistical tools for fast visualization and analysis of expression and genomic structural variation data

*Website:*http://www.chem.agilent.com/en-US/products/software/lifesciencesinformatics/genespringgx/pages/gp34525.aspx

*License needed:*Yes

Name: ANAIS

*Comment:*User-friendly web-based tool for the processing of NimbleGen expression data

*Website:*http://anais.versailles.inra.fr/

*License needed:*No

Name: JMP

*Comment:*Software for Microarray, SNP and Proteomics Expression Analysis

*Website:*http://www.jmp.com/software/webinars/jmp_microarray.shtml

*License needed:*Yes

Name: SAM: Significance Analysis of Microarrays

*Comment:*Supervised learning software for genomic expression data mining

*Website:*http://www-stat.stanford.edu/~tibs/SAM/

*License needed:*No

Name: GenePublisher

*Comment:*Gene expression analysis

*Website:*http://www.cbs.dtu.dk/services/GenePublisher/

*License needed:*No

#### B. Next Generation Sequencing Data Analysis

Blog

Name: SEQanswers

*Comment:*SEQanswers is a blog founded to be an information resource and user-driven community focused on all aspects of next-generation genomics. A reasonably thorough table of next-gen-seq software available in the commercial and public domain is provided

*Website:*http://seqanswers.com/forums/showthread.php?t=43

Note:

Examples

Name: Cufflinks

*Comment:*Cufflinks assembles transcripts, estimates their abundances, and tests for differential expression and regulation in RNA-Seq samples. It accepts aligned RNA-Seq reads and assembles the alignments into a parsimonious set of transcripts, free of charge

*Website:*http://cufflinks.cbcb.umd.edu/

*License needed:*No

*Name*: *Bowtie*

*Comment:*Bowtie is an ultrafast, memory-efficient short read aligner. It aligns short DNA sequences (reads) to the human genome at a rate of over 25 million 35-bp reads per hour. Bowtie indexes the genome with a Burrows-Wheeler index to keep its memory footprint small: typically about 2.2 GB for the human genome (2.9 GB for paired-end).

*Website:*http://bowtie-bio.sourceforge.net/index.shtml

*License needed:*No

*Name*: *Tophat*

*Comment:*TopHat is a program that aligns RNA-Seq reads to a genome in order to identify exon-exon splice junctions. It is built on the ultrafast short read mapping program Bowtie. TopHat runs on Linux and OS X.

*Website:*http://tophat.cbcb.umd.edu/

*License needed:*No

Name: Oases

*Comment:*Oases is a *de novo *transcriptome assembler designed to produce transcripts from short read sequencing technologies, such as Illumina, SOLiD, or 454 in the absence of any genomic assembly

*Website:*http://www.ebi.ac.uk/~zerbino/oases/

*License needed:*No

Name: Genomatix Genome Analyzer (GGA) and Genomatix Software Suite (GSS)

*Comment:*GGA comprehensive second-level analysis of Next Generation Sequencing (NGS) data from ChIP-Seq, RNA-Seq or genotyping experiments. GSS conducts a scientific analysis of genomic data in gene regulation, networks, pathways and genome annotation visualization.

*Website:*http://www.genomatix.de/en/index.html

*License needed:*yes

Name: ALLPATHS-LG

*Comment:*genome assembly algorithms recommended by the Broad Institute

*Website:*http://www.broadinstitute.org/science/programs/genome-biology/computational-rd/computational-research-and-development

*License needed:*no

#### C. Multicolor Cytometric Data-Analysis

Examples

Name: FlowJo

*Comment:*FlowJo is designed around the structure of flow data and the researcher's experiments. Through FlowJo's patent-pending Groups structure, for example, gates can be applied to many samples as easily as one

*Website:*http://www.treestar.com

*License needed:*Yes

Name: WinList/FCOM

*Comment:*Load FCS files from instruments and do FACS data analysis with a full set of region tools and gates. The algorithms allow for rapid generation of registers with frequencies/numbers of all possible suphenotype combinations. Can be used to input data for cluster analysis and heat maps, allowing rapid visualization of numerous complex data sets.

*Website:*http://www.vsh.com/

*License needed:*Yes

Name: FCSPress

*Comment:*FCSPress is an easy-to-use Macintosh program that produces presentation quality graphics and generates statistics from flow-cytometric data

*Website:*http://www.fcspress.com/

*License needed:*Yes

Name: FCS EXpress

*Comment:*FCS Express is designed to bring the power sophisticated analysis protocols to users in an intuitive, easy to grasp manner

*Website:*http://www.denovosoftware.com/

*License needed:*Yes

Name: GemStone

*Comment:*GemStone is software for analysis of high-dimensional, flow cytometry data. Based on patented Probability State Modeling technology, GemStone eliminates some problems that have faced flow cytometry. Subjective gating and associated errors are eliminated. Population overlaps in multidimensional data are accounted for. Multiple samples may be combined into one coherent analysis

*Website:*http://www.vsh.com/

*License needed:*Yes

Name: SPICE

*Comment:*SPICE is a data mining software application that analyzes large FLOWJO data sets from polychromatic flow cytometry and organizes the normalized data graphically. SPICE enables users to discover potential correlations in their experimental data within complex data sets.

*Website:*http://exon.niaid.nih.gov/spice/

*License needed:*Yes

### 7. Software and Tools for Function and Pathway Analysis

Examples

Name: Ingenuity Pathway Analysis (IPA)

*Comment:*User friendly software that allows analysis of biological and chemical systems

*Website:*http://www.ingenuity.com/

*License needed:*Yes

Name: GeneGo MetaCore

*Comment:*User friendly software that allows analysis of biological and chemical systems

*Website:*http://www.genego.com/about.php

*License needed:*Yes

Name: Ariadne Pathway Studio

*Comment:*Analysis of biological system with an interactive software interface and the computational approach to generating database content from the literature

*Website:*http://www.ariadnegenomics.com/

*License needed:*Yes

Name: DAVID

*Comment:*Analysis of biological system

*Website:*http://david.abcc.ncifcrf.gov/content.jsp?file=functional_annotation.html#intro

*License needed:*No

Name: KEGG Pathway

*Comment:*Collection of manually drawn pathway maps representing knowledge on the molecular interaction and reaction networks, free of charge

*Website:*http://www.genome.jp/kegg/pathway.html

*License needed:*No

Name: BioCarta pathway

*Comment:*BioCarta pathway provides displays of gene interactions within pathways for human cellular processes, such as apoptosis and signal transduction

*Website:*http://cgap.nci.nih.gov/Pathways/BioCarta_Pathways

*License needed:*No

Name: Interactive Genomics Viewer

*Comment:*The Integrative Genomics Viewer (IGV) is a high-performance visualization tool for interactive exploration of large, integrated datasets. It supports a wide variety of data types including sequence alignments, microarrays, and genomic annotations

*Website:*http://www.broadinstitute.org/igv/home

*License needed:*No

Name: GSEA

*Comment:*Gene Set Enrichment Analysis (GSEA) is a computational method that determines whether an a priori defined set of genes shows statistically significant, concordant differences between two biological states (e.g., phenotypes)

*Website:*http://www.broadinstitute.org/gsea/

*License needed:*No

Name: GOMiner

*Comment:*A tool for biological interpretation of 'omic' data - including data from gene expression microarrays. Omic experiments often generate lists of dozens or hundreds of genes that differ in expression between samples

*Website:*http://discover.nci.nih.gov/gominer/index.jsp

*License needed:*No

Name: MatchMiner

*Comment:*Set of tools that enables the user to translate between disparate ids for the same gene. It uses data from the UCSC, LocusLink, Unigene, OMIM, Affymetrix and Jackson data sources to determine how different ids relate. Supported id types include, gene symbols and names, IMAGE and FISH clones, GenBank accession numbers and UniGene cluster ids

*Website:*http://discover.nci.nih.gov/matchminer/index.jsp

*License needed:*No

Name: PANTHER Classification System

*Comment:*Panther is a resource that classifies genes by their functions, using published scientific experimental evidence and evolutionary relationships to predict function even in the absence of direct experimental evidence. Proteins are also classified

*Website:*http://www.pantherdb.org/

*License needed:*Yes

### 8. siRNA Libraries

Examples

Name: Ambion siRNA Libraries

*Comment:*siRNA Libraries

*Website:*http://www.ambion.com/techlib/tn/116/11.html

Name: Thermo Scientific siRNA Libraries

*Comment:*siRNA Libraries

*Website:*http://www.dharmacon.com/catalog/catalogitemtemplate.aspx?id=1435&imageid=1882

Name: siRNA Libraries

*Comment:*siRNA Libraries

*Website:*http://genome.duke.edu/cores/rnai/libraries/qiagen/

### 9. Public Databases

Name: NCBI Databases

*Comment:*Gene/Protein/SNP/Nucleotide and several other databases

*Website:*http://www.ncbi.nlm.nih.gov/gquery/

Name: Gene Expression Omnibus (GEO)

*Comment:*Public functional genomics data repository supporting MIAME-compliant (Minimum information about a microarray experiment) data submissions. Array- and sequence-based data are accepted. Tools are provided to help users query and download experiments and curated gene expression profiles

*Website:*http://www.ncbi.nlm.nih.gov/geo/

Name: Catalogue Of Somatic Mutations In Cancer (COSMIC)

*Comment:*COSMIC is designed to store and display somatic mutation information and related details and contains information related to human cancers

*Website:*http://www.sanger.ac.uk/genetics/CGP/cosmic/

Name: The Cancer Genome Atlas (TCGA)

*Comment:*The Cancer Genome Atlas (TCGA) Data Portal provides a platform for researchers to search, download, and analyze data sets generated by TCGA. Launched in 2006 as a partnership between the National Cancer Institute and the National Human Genome Research Institute, both NIH components, The Cancer Genome Atlas (TCGA) has developed a comprehensive strategy for comparing the genome of cancer cells to the genome of normal cells from the same patient

*Website:*http://cancergenome.nih.gov/

Name: The Human Protein Atlas

*Comment:*The Human Protein Atlas portal is a publicly available database with millions of high-resolution images showing the spatial distribution of proteins in 46 different normal human tissues and 20 different cancer types, as well as 47 different human cell lines. The data is released together with application-specific validation performed for each antibody. The database was developed in a gene-centric manner with the inclusion of all human genes predicted from genome efforts

*Website:*http://www.proteinatlas.org

Name:DIP (Database of Interacting Proteins)

Comment:The DIP database catalogs experimentally determined interactions between proteins. It combines information from a variety of sources to create a single, consistent set of protein-protein interactions

Website:http://dip.doe-mbi.ucla.edu/dip/Main.cgi

Name:MINT (Molecular INTeraction Database)

Comment:MINT focuses on experimentally verified protein-protein interactions mined from the scientific literature by expert curators. The curated data can be analyzed in the context of the high throughput data and viewed graphically with the 'MINT Viewer'

Website:http://mint.bio.uniroma2.it/mint/Welcome.do

Name: Human Protein Reference Database

*Comment:*The Human Protein Reference Database represents a centralized platform to visually depict and integrate information pertaining to domain architecture, post-translational modifications, interaction networks and disease association for each protein in the human proteome

*Website:*http://www.hprd.org

Name: Cancer Genome Anatomy Project (CGAP)

*Comment:*The NCI's Cancer Genome Anatomy Project sought to determine the gene expression profiles of normal, precancer, and cancer cells, leading eventually to improved detection, diagnosis, and treatment for the patient. Resources generated by the CGAP initiative are available to the broad cancer community

*Website:*http://cgap.nci.nih.gov/cgap.html

Name: caBIG

*Comment:*caBIG stands for the cancer Biomedical Informatics Grid. caBIG is an information network enabling members of the cancer community - researchers, physicians, and patients - to share data and knowledge

*Website:*https://cabig.nci.nih.gov/

Name: mirbase

*Comment:*Searchable database of published miRNA sequences and annotation

*Website:*http://www.mirbase.org

Name: deepBase

*Comment:*deepBase is a database for annotating and discovering small and long ncRNAs (microRNAs, siRNAs and piRNAs) from high-throughput deep sequencing data

*Website:*http://deepbase.sysu.edu.cn/miRDeep.php

Name: Gene Ontology Annotation (UniProtKB-GOA) Database

*Comment:*The UniProtKB-GOA project aims to provide high-quality Gene Ontology (GO) annotations to proteins in the UniProt Knowledgebase (UniProtKB) and International Protein Index (IPI) and is a central dataset for other major multi-species databases; such as Ensembl and NCBI

*Website:*http://www.ebi.ac.uk/GOA/

Name: Protfun

*Comment:*The ProtFun 2.2 server produces *ab initio *predictions of protein function from sequence

*Website:*http://www.cbs.dtu.dk/services/ProtFun/

Name: TRANSFAC 7.0 Public 2005

*Comment:*Data on transcription factors, their experimentally-proven binding sites, and regulated genes. Its broad compilation of binding sites allows the derivation of positional weight matrices

*Website:*http://www.gene-regulation.com/pub/databases.html

Name: OptiTope

*Comment:*OptiTope aims at assisting immunologists in designing epitope-based vaccines. It is an easy-to-use tool to determine a provably optimal set of epitopes with respect to overall immunogenicity in a specific individual or a target population, free of charge

*Website:*http://www.epitoolkit.org/optitope

Name: Cancer Central Clinical Database (C3D)

*Comment:*Cancer Central Clinical Database (C3D) is a clinical trials data management system. C3D collects clinical trial data using standard case report forms (CRFs) based on common data elements (CDEs)

*Website:*https://cabig.nci.nih.gov/tools/c3d

Name: UCSC Genome Browser

*Comment:*Provides a large database of publicly available sequence and annotation data along with an integrated tool set for examining and comparing the genomes of organisms, aligning sequence to genomes, and displaying and sharing users' own annotation data

*Website:*http://genome.ucsc.edu/

Name: Next Bio

*Comment:*Exhaustive collection of public microarray data. NextBio's platform combines powerful tools with unique correlated content. With NextBio you can search tens of thousands of studies containing billions of data points spanning different experimental platforms, organisms and data types

*Website:*http://www.nextbio.com/b/corp/faq.nb

Name: SYFPEITHI

*Comment:*SYFPEITHI is a database comprising more than 7,000 peptide sequences known to bind class I and class II MHC molecules. The entries are compiled from published reports only

*Website:*http://www.syfpeithi.de/

Name: Melanoma Molecular Map Project

*Comment:*MMMP Databases, putting together the pieces of the melanoma puzzle. Seven interconnected databanks for the interactive collection, update and consultation of the translational and clinical information on melanoma biology and treatment.

*Website:*http://www.mmmp.org

Name: The Targeted Therapy Database (TTD)

*Comment:*Systematic collection of the scientific knowledge regarding the development of targeted therapy for melanoma

*Website:*http://www.mmmp.org

### 10. Tools for the Bench and Other Useful Websites

#### A. Primer Design Software

Examples

Name: Primer3

*Comment:*Primer3 is a free online tool to design and analyze primers for PCR and real time PCR experiments. Primer3 can also select single primers for sequencing reactions and can design oligonucelotide hybridization probes

*Website:*http://frodo.wi.mit.edu/primer3

Name: Oligo Analyzer 3.0

*Comment:*Software developed by IDT (Integrated DNA Technologies) that analyzes physical properties of a specific oligo sequence. The results show a complementary sequence, oligo length, GC content, melting temperature, extinction coefficient, molecular weight, μg/OD, and nmoles/OD.

*Website:*http://www.idtdna.com/analyzer/applications/oligoanalyzer

Name: MethPrimer

*Comment:*MethPrimer is a program for designing bisulfite-conversion-based methylation PCR Primers. It can design primers for Methylation-Specific PCR (MSP), Bisulfite-Sequencing PCR (BSP) and Bisulfite-Restriction PCR

*Website:*http://www.urogene.org/methprimer/index1.html

#### B. Transcription Factors Binding Sites Prediction Software

Examples

Name: TFSEARCH

*Comment:*TFSEARCH predicts transcription factors binding sites from a given sequence. It does simple correlation calculation with binding site profile matrices

*Website:*http://www.cbrc.jp/research/db/TFSEARCH.html

#### C. Design of Antisense Oligonucleotides, Nucleic Acid Probes, siRNA Software

Name: Sfold

*Comment:*Sfold predicts RNA duplex thermodynamics for rational siRNA design. It supports target accessibility prediction and rational design of antisense oligonucleotides (ASO) and nucleic acid probes. It can design an ASO for a gene of interest based on the mRNA sequence

Website:http://sfold.wadsworth.org/cgi-bin/soligo.pl

#### D. miRNA Prediction

Examples

Name: TargetScan

*Comment:*Prediction of biological targets of miRNAs

*Website:*http://www.targetscan.org/

*License needed:*No

*Name:*http://microRNA.org

*Comment:*A resource for predicted microRNA targets and expression, free of charge

*Website:*http://www.microrna.org/microrna/home.do

*License needed:*No

Name: DIANA LAb

*Comment:*miRNA computational predictive models. Experimental supported databases

*Website:*http://diana.cslab.ece.ntua.gr/?sec=home

*License needed:*No

Name: RNA22

Comment:This software first finds putative microRNA binding sites in the sequence of interest, then identifies the targeting microRNA

Website:http://cbcsrv.watson.ibm.com/rna22.html

Name: PicTar

*Comment:*PicTar provides combinatorial microRNA target predictions

*Website:*http://pictar.mdc-berlin.de

Name: miRANDA

*Comment:*miRanda is an algorithm for finding genomic targets for microRNAs

*Website:*http://www.microrna.org/microrna/home.do

#### E. Alternative Splicing Analysis

Example

Name: SpliceCenter

*Comment:*The SpliceCenter applications are user-friendly tools that provide information on the target location of probesets, primers, or siRNAs within the known splice variants of a gene

*Website:*http://www.tigerteamconsulting.com/SpliceCenter/

*License needed:*No

#### F. Linkage Disequilibrium Analysis

Examples

Name: PhAT

*Comment:*It analyzes SNP data, showing pairwise Linkage Disequilibrium of various types (r^2^, r, D' and abs(D)) and producing graphical matrices of obtained results

*Website:*http://pharmgat.org/Tools/pbtoldplotform

#### G. Analysis Support, Laboratory Optimization and Other Useful Websites

Examples

Name: Immuneering

*Comment: Computer Model*. Development of computer models that aim to predict the response to biological therapies in cancer patients

*Website:*http://www.immuneering.com/

Name: Cellumen CellCiphr Patient Sample Profiling

*Comment:*To use the cellular systems biology approach to improve patient stratification for clinical trial enrollments. Cellumen is collaborating with the Mayo Clinic and Foundation to create panels of cellular biomarkers using multiplexed fluorescence to apply to patient cells and tissues, starting with breast cancer

*Website:*http://www.cellumen.com/solutions/patient.php

Name: Biotracker

*Comment: Lab Management*. Biotracker is a specialty Lab Information Management Solution (LIMS) for enhancing productivity and effectiveness in life sciences research laboratories

*Website:*http://www.ocimumbio.com/lims2/products/

Name: Gene's Logic Expression Array Analysis

*Comment:*Array analysis support; team dedicated to statistical and bioinformatic analyses of microarray data, ranging from basic quality control and differential gene expression analysis

*Website:*http://www.genelogic.com/services/bioinformatic-analysis

Name: Microsoft Word Add-In for the GenePattern Reproducible Research Document (GRRD)

*Comment:*To facilitate publishing reproducible results, GenePattern automatically captures the history of any computational work being done, allowing scientists to easily generate pipelines to reproduce computational methods

*Website:*http://www.broadinstitute.org/cancer/software/genepattern/grrd/AddIn.html

*License needed:*No

Name: IUBio Archive

*Comment:*Archive of biology data and software, established in 1989 to promote public access to freely available information, primarily in the field of molecular biology and bioinformatics

*Website:*http://iubio.bio.indiana.edu/

Name: Labome

*Comment:*Tools for searching antibodies, siRNA/shRNA, ELISA, cDNA clones, proteins/peptides, microRNA, and biochemicals from all suppliers

*Website:*http://www.labome.com/

*License needed:*No

#### H. Nanotechnology

Examples

Name: Inno.CNT

*Comment:*The Inno.CNT website gives an updated and wide overview of the research status of carbon nanotubes as one of the most promising nanomaterial to open up completely new dimensions in biomedical applications

*Website:*http://www.inno-cnt.de/en/uebercnt.php

Name: ObservatoryNano

*Comment:*The ObservatoryNano provides wide-ranging analysis focusing on nanoscience and nanotechnology developments

*Website:*http://www.observatory-nano.eu/project/

Name: Nanofutures

*Comment:*Nanofutures is an integration platform aimed at becoming a long-lasting nanotechnology hub, connecting the most relevant nanotechnology stakeholders

*Website:*http://www.nanofutures.eu/

Name: Nanotechnology Medicine

*Comment:*Nanotechnology Medicine brings you informative nanomedicine articles, educational nanomedicine videos, and lively nanomedicine dialogue and chatter.

*Website*: http://nanotechnologymedicine.org/

#### I. Clinical Trials Registries

Example

Name: Clinical Trial Registries

*Comment:*The five clinical trials registries approved by the International Committee of Medical Journal Editors (ICMHE)

*Website:*http://ClinicalTrials.gov, http://www.actr.org.au, http://www.ISRCTN.org, http://www.umin.ac.jp/ctr/index/htm, http://www.trialregister.nl

## Conclusion

Immune biomarkers are playing an increasingly important role in the successful development, clinical evaluation, and immune monitoring of cancer immunotherapies. The references, products and online resources in this Cancer Immunotherapy Biomarkers Resource Document were identified by the authors and the SITC/iSBTc Taskforce on Immunotherapy Biomarkers to support the discovery, evaluation and application of biomarkers for cancer immunotherapy. These selected references and links serve as a compass to point investigators to useful resources in this ever growing, and important field of cancer immunotherapy biomarkers. Emerging issues surrounding cancer immunotherapy biomarker discovery and clinical application will continue to be addressed in upcoming SITC Annual Meetings and Associated Programs [13].

## Competing interests

DB, JB, EW, MLD, CMB, LGD, ST, TFG, LHB and FFM declare that they have no competing interests. BAF is co-founder of UbiVac, LLC and serves on the scientific advisory boards of Micromet, Inc. and MannKind Corporation.

## Authors' contributions

DB and JB prepared the manuscript. EW, MLD, CMB, LGD, ST, BAF, TFG, FMM and LHB provided substantive editing and critical review. All authors read and approved the final manuscript.

## References

[B1] Symposium on Immuno-Oncology Biomarkers, 2010 and Beyond: Perspectives from the iSBTc Biomarker Task Force http://www.sitcancer.org/meetings/am10/biomarkers10/10.1186/1479-5876-8-130 PMC3014892 21138581

[B2] SITC/iSBTc-FDA-NCI Workshop on Prognostic and Predictive Immunologic Biomarkers in Cancer http://www.sitcancer.org/meetings/am09/workshop09/

[B3] Immune Monitoring Workshop II http://www.sitcancer.org/meetings/am04/workshop.php

[B4] Workshop on Cancer Biometrics: Identifying Biomarkers and Surrogates of Tumors in Patients http://www.sitcancer.org/meetings/am03/biometrics.php 10.1097/01.cji.0000154251.20125.2e 15725954

[B5] Immune Monitoring Workshop http://www.sitcancer.org/meetings/bethesda/immune_monitoring_workshop.php

[B6] Butterfield LH Palucka AK Britten CM Dhodapkar MV Hakansson L Janetzki S Kawakami Y Kleen TO Lee PP Maccalli C Maecker HT Maino VC Maio M Malyguine A Masucci G Pawelec G Potter DM Rivoltini L Salazar LG Schendel DJ Slingluff CL Jr Song W Stroncek DF Tahara H Thurin M Trinchieri G van Der Burg SH Whiteside TL Wigginton JM Marincola F Khleif S Fox BA Disis ML Recommendations from the iSBTc-SITC/FDA/NCI Workshop on Immunotherapy Biomarkers Clin Cancer Res 2011 17 3064 3076 10.1158/1078-0432.CCR-10-2234 21558394 PMC3096674

[B7] Butterfield LH Disis ML Khleif SN Balwit JM Marincola FM Immuno-Oncology Biomarkers 2010 and Beyond: *Perspectives from the iSBTc/SITC Biomarker Task Force* J Transl Med 2010 8 1 130 10.1186/1479-5876-8-130 21138581 PMC3014892

[B8] Butterfield LH Disis ML Fox BA Lee PP Khleif SN Thurin M Trinchieri G Wang E Wigginton J Chaussabel D Coukos G Dhodapkar M Håkansson L Janetzki S Kleen TO Kirkwood JM Maccalli C Maecker H Maio M Malyguine A Masucci G Palucka AK Potter DM Ribas A Rivoltini L Schendel D Seliger B Selvan S Slingluff CL Jr Stroncek DF Streicher H Wu X Zeskind B Zhao Y Zocca MB Zwierzina H Marincola FM A systematic approach to biomarker discovery; Preamble to "the iSBTc-FDA taskforce on immunotherapy biomarkers" J Transl Med 2008 6 81 10.1186/1479-5876-6-81 19105846 PMC2630944

[B9] Lotze MT Wang E Marincola FM Hanna N Bugelski PJ Burns CA Coukos G Damle N Godfrey TE Howell WM Panelli MC Perricone MA Petricoin EF Sauter G Scheibenbogen C Shivers SC Taylor DL Weinstein JN Whiteside TL Workshop on cancer biometrics: identifying biomarkers and surrogates of cancer in patients J Immunother 2005 28 79 119 10.1097/01.cji.0000154251.20125.2e 15725954

[B10] Keilholz U Weber J Finke J Gabrilovich D Kast WM Disis N Kirkwood JM Scheibenbogen C Schlom J Maino VC Lyerly HK Lee PP Storkus W Marincola F Worobec A Atkins MB Immunologic monitoring of cancer vaccine therapy: results of a Workshop sponsored by the Society of Biological Therapy J Immunother 2002 25 97 138 10.1097/00002371-200203000-00001 12074049

[B11] Brazas MD Yamada JT Ouellette BFF Providing web servers and training in Bioinformatics: 2010 update on the Bioinformatics Links Directory Nucleic Acids Res 2010 38 Web Server W3 W6 10.1093/nar/gkq553 20542914 PMC2896181

[B12] Bioinformatics Links Directory http://bioinformatics.ca/links_directory/journals/nar/2010

[B13] SITC 2011 Annual Meeting and Associated Programs http://www.sitcancer.org/meetings/

